# “Unless they bring it up, I won't go digging”: Psychiatric nurses' experiences of developing therapeutic relationships with adult survivors of child sexual abuse

**DOI:** 10.1111/ppc.13085

**Published:** 2022-04-05

**Authors:** Chloe Walsh, Stephen K. Bradley, John Goodwin

**Affiliations:** ^1^ Cork/Kerry Mental Health Services HSE South, HSE Cork Ireland; ^2^ Faculty of Lifelong Learning IT Carlow Carlow Ireland; ^3^ Catherine McAuley School of Nursing and Midwifery University College Cork Cork Ireland

**Keywords:** child sexual abuse, CSA, qualitative research, therapeutic relationships

## Abstract

**Purpose:**

The purpose of this study is to explore psychiatric nurses' experiences of developing therapeutic relationships with adult survivors of child sexual abuse (CSA).

**Design and Methods:**

A qualitative descriptive design was adopted. Semistructured interviews were conducted with six registered psychiatric nurses. Data were analyzed using reflexive thematic analysis.

**Findings:**

Although participants were able to develop therapeutic relationships with survivors and cited the importance of interpersonal skills, they felt uncomfortable discussing CSA.

**Practice Implications:**

Given the importance of developing trusting relationships, more support needs to be provided for nurses so they can build stronger alliances with survivors of CSA.

## 1 INTRODUCTION

Child sexual abuse (CSA) is defined as when a child is used by a perpetrator (or perpetrators) for the purpose of sexual arousal through direct contact or noncontact abuse (National Society of Prevention of Cruelty to Children, 2021). The World Health Organization, 2017) has indicated that CSA is a violation of human rights and a major international public health concern. CSA has affected 120 million children, globally (UNICEF, 2017), with 20% of girls and 14% of boys under the age of 18 years having been victims of sexual abuse (Moody et al., 2018).

As a result of CSA, children can experience adverse neurobiological, physical, psychological, sexual, and social effects (Azzopardi et al., 2021; WHO 2017), including emotional dysregulation, sleep disturbances (Demirci, 2018), substance misuse, depression, and suicidal ideation (Gray & Rarick, 2018). Consequently, many victims of CSA require mental health intervention. Although CSA victims will access mental health supports as children/adolescents (Azzopardi et al., 2021; WHO 2017), given the long‐term effects of CSA, many of these victims become users of the adult mental health services (Kennedy et al., 2021; MacIntosh & Ménard, 2021). Indeed, it has been estimated that 30% of people presenting to mental health services have experienced some form of sexual abuse (Health Services Research Centre, 2003).

Within the mental health services, the development of therapeutic relationships with staff is paramount for victims of CSA (Azzopardi et al., 2021; Sivagurunathan et al., 2019). Strong therapeutic relationships between healthcare practitioners and service users have a significant impact on recovery and are the greatest predictor of therapeutic outcome (Duncan et al., 2010). Adult survivors of CSA have indicated that engaging in therapeutic processes and building trusting relationships with staff members is transformative as it facilitates the start of their healing journeys (Easton et al., 2015).

Although developing therapeutic relationships with service users is the primary role of the psychiatric nurse (Wright & McKeown, 2018) evidence suggests that these healthcare practitioners are uncomfortable addressing issues surrounding CSA (Keltner & Steele, 2018; Kennedy et al., 2021). Keltner and Steele (2018) discuss personal implications for healthcare professionals working with adult survivors of CSA, acknowledging the possibility of secondary posttraumatic stress disorder, which may lead to burn out.

However, it is important that psychiatric nurses develop confidence around supporting adult victims of CSA. In several counties, it is now mandatory by law for mandated persons (including healthcare practitioners) to abide by legislation regarding retrospective disclosures by adults (Her Majesty's Government, 2021; Tusla, 2017). Psychiatric nurses also need to consider that over half of CSA victims experience revictimization in later life (Papalia et al., 2021), and that between 3% and 36% of people have experienced sexual assaults while in mental healthcare (Barnett, 2020; Rossa‐Roccor et al., 2020). The promotion of reflection and self‐awareness amongst psychiatric nurses is a key component and skill in providing therapeutic support for survivors of CSA (Leech & Trotter, 2006) and psychiatric nurses need to be mindful of how they respond to survivors (Su & Stone, 2020). To enhance care and develop effective therapeutic policies for survivors of CSA, it is vital that the insights from healthcare practitioners, including nurses, are considered (Sivagurunathan et al., 2019).

## 2 PURPOSE

The aim of this study is to explore psychiatric nurses' experiences of working with adult survivors of CSA and the development of therapeutic relationships with these individuals.

## 3 DESIGN AND METHODS

This study was guided by a qualitative descriptive approach. Rather than providing evidence to support existing theories, the aim of a qualitative descriptive study is to understand and describe phenomena. This involves staying close to the data and taking an inductive approach to analysis (Bradshaw et al., 2017).

### 3.1 Recruitment and participants

A purposeful convenience sampling approach was adopted. A poster advertising the study was displayed in prominent locations in local acute admissions units in a health service in Cork, Ireland. Potential participants were invited to contact the lead researcher directly.

### 3.2 Data collection

Participants provided informed consent before their individual interview. Interviews were guided by a semistructured interview guide, informed by a review of the literature. Participants were asked questions about their experiences of engaging with survivors of CSA, and to identify the facilitators and the barriers to engaging in therapeutic relationships with survivors. Follow‐up prompts were also used to promote discussion. Interviews were audiorecorded and transcribed verbatim by the lead author. Once transcription was completed, all recordings were destroyed.

### 3.3 Data analysis

Data were analyzed using Braun and Clarke's (2019) reflexive thematic analysis. This is an approach to analyzing qualitative data that encourages researcher reflexivity when interpreting people's subjective experiences (Braun & Clarke, 2021). Immersion was facilitated by the lead researcher listening to the audio recordings several times, before transcription. Transcripts were initially coded, which involved condensing/shortening the text into manageable data while ensuring that the core meaning was retained (Graneheim & Lundman, 2004). Coding was initially completed by CW and cross‐checked by JG. From these codes, initial themes were then generated by CW. These themes were debated, discussed, and refined.

Rigor was guided by Lincoln and Guba's (1985) principles of credibility, dependability, confirmability, and transferability. Credibility was established by the lead researcher/interviewer expressing compassion and empathy during interviews, as recommended by Bradshaw et al. (2017). Furthermore, the researcher who conducted the interviews was familiar with four of the six participants; such familiarity has been highlighted as a way to promote rapport during the research process (Bamidele et al., 2019; Cook & Larson, 2021; Naylor & Nyanjom, 2021), strengthening the credibility of the study. An audit trail was maintained throughout the research process, to enhance confirmability and dependability. A purposive sampling approach was used to enhance transferability (Bradshaw et al., 2017).

### 3.4 Ethics

This study was approved by the Clinical Research Ethics Committee and access to participants was granted by the Director of Nursing. An ethical approach to the study was guided by Beauchamp and Childress's (2013) core moral principles of autonomy, beneficence, nonmaleficence, and justice. In keeping with the principle of autonomy, it was stated on the poster that participation was entirely voluntary. Given that the aim of the current study is to explore how nurses develop therapeutic relationships with survivors of CSA, the principle of beneficence was adhered to. Participants were made to feel comfortable throughout the interviews and provided with the researchers' contact details should they experience any distress; in this regard, we adhered to the principle of nonmaleficence.

## 4 FINDINGS

Six participants volunteered to take part in the study. Pseudonyms are used to disguise participants' identities. Demographics are reported in Table 1.

**Table 1:** Demographics

Pseudonym	Years of experience	Gender	Nursing grade	Highest level of education achieved
Alan	15	Male	Staff nurse	MSc
Beth	9	Female	Staff nurse	MSc
Clara	15	Female	Clinical nurse manager, Grade 2	MSc
Diane	20	Female	Staff nurse	MSc
Emma	15	Female	Clinical nurse specialist	MSc
Faye	25	Female	Staff nurse	Diploma

Three main themes were identified from the data as follows: “A reluctance to develop therapeutic relationships with survivors of CSA,” “The process of developing therapeutic relationships”, and “Supports needed to enhance therapeutic relationships”.

### 4.1 A reluctance to develop therapeutic relationships with survivors of CSA

Participants suggested that, in the event of a CSA survivor not raising the issue of their abuse, this would not be explored by the psychiatric nurse. Exploring a history of trauma was not perceived as part of the nurse's role:

“I'd be very slow in digging into their pasts if I had no… if I didn't know what to do with it. If there was no aim for why I drilled into their pasts, which are horrific… unless they bring it up, I won't go digging” (Alan).

“I'm not a qualified counsellor in relation to dealing with the issue” (Diane).

The role of engaging therapeutically with survivors was seen as more relevant to other disciplines. One participant felt that a professional counseling service was the most appropriate intervention for a survivor under her care. However, owing to a lengthy waiting period to access this service, this service user was instead referred to the team's psychologist:

“We were recommending […] counselling, but […] the waiting list is a year‐long, so that's what we find very difficult on the crisis team, when someone discloses that they've been sexually abused, the waiting list—they may interview them, assess them, but they'll still be waiting a whole year, so it's a long time to be […] waiting, ya know, so it's great now we've got a psychologist on board” (Ella).

The reluctance participants demonstrated to discuss childhood trauma related to a lack of confidence in how to support survivors. Participants were apprehensive that opening up “old wounds” can leave survivors in a vulnerable position, but owing to perceived limitations in therapeutic skills, it may not be possible for the psychiatric nurse to address survivors' issues:

“I think nurses are a little bit afraid to open up a wound, an old wound even. That, the person start's talking about it then, and they don't know, how to comfort them then, or end‐end the 1:1 in a positive note. They feel like if I start talking about their child sexual abuse that they're after opening up this… and then they're not therapeutic enough” (Clara).

“I don't know, look I think it's going back to 'what are you gonna do with it?' If I open a can of worms, am I just being a voyeur?” (Alan).

Consequently, rather than advancing the therapeutic relationship, it was felt that nurses could actually damage their relationships with survivors by addressing the issue of CSA, forcing the survivor to confront their past but demonstrating an inability to address their experiences in a meaningful way:

“Not sure I'd be comfortable going too deeply into someone's childhood sexual abuse unless they brought it up… Sometimes the more they rehash it, I don't know is it just making it worse. […] I'm just picking scabs with nowhere to put them on to, or the next step, or they could crumble and become even worse” (Alan).

Another barrier to developing therapeutic relationships with survivors was the impact their narratives had on the mental health of nurses:

“[Working with survivors of CSA] affects your own belief system and your own mental health” (Faye).

“You do hear about, um, horrific things that have happened to people—it does change your view on the world and it makes you feel like the world is a scarier darker place, because you don't hear the good stories” (Clara).

Participants suggested that the emotional impact of engaging with CSA survivors was challenging, precluding the successful progression of the therapeutic relationship, meaning encounters with survivors is maintained at a superficial level:

“It's a tough one for maybe some nurses, to get very heavily involved with someone like that” (Beth).

“It saddens me a lot. It really does. It does influence the way I work with people, and I don't know what to do with it” (Alan).

The emotional impact of engaging with CSA survivors extended beyond the work environment. Participants reported that they sometimes felt “numb” for several days following discussions with survivors. One participant commented that one survivor's narrative continued to affect them in their personal life, as they demonstrated reluctance to engage socially with their peers:

“We [myself & colleague] had to go for coffee afterwards and a chat, we found it very traumatic actually, in our own hearts we were shaken by it really… we found it very difficult. For days afterwards I felt a bit numb. […] I remember having to go out that night socially and I didn't wanna go […] you're just human and you do take some of their problems on board” (Ella).

It was suggested that the reluctance to develop a therapeutic relationship was bidirectional. Before survivors felt comfortable in addressing their trauma, it was noted that trust and rapport would need to be developed:

“I suppose from previous experience of working with these people, they need to build up a bit of trust and rapport with a person before they disclose something like that. So, if you're just admitting them and it's your first time possibly meeting them and you'd say, ‘Oh, any, you know, past, ya know, history of abuse', they're gonna say 'no' because they don't know you from Adam and feel uncomfortable at that time” (Beth).

### 4.2 The process of developing therapeutic relationships

Participants felt that developing trust is the foundation for a successful therapeutic relationship. To build trust, the importance of confidentiality was highlighted, with participants emphasizing the need to be very explicit about this from the beginning with CSA survivors:

“I think, you know, somebody isn't probably going to disclose something if they don't feel, that it's going to be, confidential environment anyway and that they can trust the person that they're telling and that” (Diane).

“I think the attitude is very important really to, maybe, have a positive attitude, and just to tell people in the beginning that it is very confidential, […] just to have a very trusting, therapeutic relationship with them” (Ella).

Key interpersonal and communication skills, such as active listening and being empathetic were regarded as central to fostering therapeutic relationships. Although participants commented that it was important to ask survivors questions, caution was advised about pushing boundaries:

“Listening. Get good at listening. […] Supporting. Generally, talking about, how it's affected them, […] without prying too much” (Alan).

“I think, as well, it's about really building up a trusting, um, making people comfortable, with you as well, having empathy. […] In the beginning, it's important to listen, and maybe to, um, prompt people to say things but not necessarily ask them too many questions, I think is key” (Ella).

When exploring survivors' narratives, participants felt that nurses needed to be conscious about giving time to the people under their care. It was felt that conversations around trauma need to be guided by survivors and the pace at which they chose to disclose events from their past needs to be respected:

“Giving the person time, um, allowing them to talk about things, in their own time […] and allowing them to disclose at their pace as well and not putting any pressure on them—that can be a big thing” (Diane).

Participants also highlighted the importance of being nonjudgmental when engaging therapeutically with survivors. It was suggested that preconceptions needed to be put aside to enhance meaningful therapeutic relationships and to adopt a person‐centered approach:

“And nurses, I suppose, to be aware of their conditioning, aware of where they're coming from, aware if they are judgemental, aware… aware of their baggage, so they can just be as helpful as they can to someone” (Alan).

However, several participants commented that, owing to frequent service use and multiple presentations, the trauma survivors have experienced is often forgotten. It was suggested that, when working therapeutically with survivors, psychiatric nurses sometimes focused on current presentations and symptoms, overlooking contextual factors, such as the sexual abuse they have experienced:

“But, say if a service user has been in the services for many years, many presentations, many admissions, I think, people seem to forget that. And especially if they're a challenging service user, […] their history is very much forgotten” (Alan).

“I feel that people do feel empathy and they do, you know, they would feel that's a terrible thing, but sometimes because it's so historical, they, they're not thinking about that. You know, they're thinking about the behaviour right in front of them” (Clara).

Despite these factors being overlooked, participants acknowledged the importance of addressing the trauma in survivors' past. Participants recommended that, rather than concentrating on diagnoses and the labels assigned to survivors, nurses should instead consider the traumatic circumstances underscoring their mental distress:

“If you hear somebody has had, um, a background of sexual abuse, I think there's more, more liable you're more liable to feel more empathy or sympathy towards them rather than if you hear first of all, ‘actually this person's diagnosis is emotionally unstable personality disorder or and by the way they were abused as a child' kind of thing” (Faye).

“I suppose it's trying to remember that the trauma this person has gone through before and like that, ya know, really, that they are, um, they have suffered or they've had trauma to a horrible degree, and, like, that that they don't know how to cope or survive. And, um, I think that that can help‐you have to bring that back though” (Clara).

### 4.3 Supports needed to enhance therapeutic relationships

Participants felt that they needed support in caring for survivors of CSA, and that they needed to build on the skills needed to develop therapeutic relationships. It was suggested that specific training around CSA would mitigate apprehension, empower nurses, and provide them the confidence they needed to work effectively with survivors:

“Some form of training, even a day course or something, […] refresh their knowledge, […] giving the mental health nurses the tools to do the right thing by the survivors” (Beth).

“Training does increase your confidence to be able to deal with and communicate with people who would divulge that kind of information and to be able to react in a supportive and encouraging way” (Faye).

“Training would help, it would give the foundation of how to work with someone [who experienced CSA]” (Alan).

It was felt that this training should be introduced in education settings for undergraduate student nurses. However, participants suggested that further training is also warranted for staff nurses:

“I really do think it should be something that is brought in, I suppose possibly brought in at an undergrad level, um, for the students to learn as part of their training, and for the qualified staff, you know, there should be some form of training provided to us” (Beth).

Given the impact of working therapeutically with survivors on nurses' mental health, the benefits of clinical supervision were discussed. Participants noted that there are no opportunities to engage in clinical supervision in some settings:

“I think supervision would be really important as well too, just so that you would have a safe environment that you could discuss things […] group supervision or individual supervision” (Diane).

However, one participant stated that the process of clinical supervision is available in their area of employment. This participant availed of this process following some challenging encounters with survivors and commented that it was beneficial to their own mental health:

“I'm okay now 'coz we've got clinical supervision once a month, so, we can discuss, your issues at clinical supervision, that helps as well so, I'm okay now” (Ella).

It was highlighted that discourses around CSA within society are beginning to change, meaning the general public are more informed, and survivors are becoming more willing to speak about their traumatic experiences. Participants suggested that such change will encourage survivors to access appropriate services and become more open to speaking to healthcare workers in future:

“I think the more information that's out there in the general public really about the fact that it's making giving people the message that it's never too late to, um, disclose information, that there's always help available and that there's no kind of expiry date on it” (Faye).

## 5 DISCUSSION

The aim of this study was to explore psychiatric nurses' experiences of working with adult survivors of CSA and the development of therapeutic relationships with these individuals. The psychiatric nurses who partook in this study felt they did not have the requisite training to adequately address the trauma experienced by survivors of CSA, with other disciplines considered more specialized in this area. Therefore, other disciplines were perceived as having more of a role to play in addressing survivors' needs. This raises the question about how therapeutic relationships are built between survivors and nurses, given that important information is not shared and discussed. There is evidence to suggest that such of feelings of inadequacy are not unique to psychiatric nurses, with several other disciplines reporting that dealing with CSA trauma is more the remit of other disciplines (Capri et al., 2013; Muridzo et al., 2018). Although specialized training may be warranted, given the lack of clarity around staff members' roles in caring for survivors, in addition to the importance of interdisciplinary psychiatric care (Happell et al., 2020; Kilty et al., 2021), such training needs to involve all team members. Otherwise, there is the potential for “diffusion of responsibility” where team members presume that survivors' needs are addressed by other professionals.

It was recommended that training around supporting survivors could be included as part of the undergraduate curriculum. Kenny and Abreu (2015) also identified that other members of mental health multidisciplinary teams—including counselors and psychologists—lacked confidence in the area of supporting survivors of CSA. Furthermore, Kenny and Abreu (2015) addressed the importance of including training around CSA as part of undergraduate programs. This further suggests a need for interdisciplinary training, which could be introduced for all healthcare students during their undergraduate studies. It is recommended that healthcare practitioners are also given opportunities to undertake more focussed evidence‐based training, such as Skills Training in Affective and Interpersonal Regulation (MacIntosh et al., 2018).

One of the key barriers to deep engagement suggested by participants was the potential to cause harm to survivors by bringing up their past trauma. In this regard, efforts to work in an altruistic fashion may lead to psychiatric nurses failing to adequately address the needs of survivors. While it is imperative that psychiatric nurses act in an altruistic fashion (Başoğul et al., 2019; Deborah et al., 2020), nurses also need to be aware that survivors' healing journeys can be best facilitated by developing meaningful therapeutic connections with others (Azzopardi et al., 2021; Easton et al., 2015; Sivagurunathan et al., 2019). In the absence of such discussions, or where CSA is ignored, van der Zalm et al. (2015) found that nurses may actually risk causing harm to survivors. Considering how central the development of therapeutic relationships is the role of the role of the psychiatric nurse, nurses need to be made more aware of the potential positive influence they can have on survivors by exploring their trauma.

Despite a reluctance to address CSA, participants demonstrated an awareness about key interpersonal skills needed to support survivors, with empathy, active listening, and adopting a nonjudgmental approach guiding conversations. This corresponds with the extant literature (van der Zalm et al., 2015), indicating that psychiatric nurses are more prepared than they realize to engage with survivors and hold the necessary skill sets to develop meaningful therapeutic relationships. However, the perceived lack of confidence around using these skills needs to be addressed. It is recommended that interdisciplinary training around CSA focuses on key interpersonal skills, with a view towards developing nurses' confidence in employing these, thus preparing them for difficult conversations with survivors of CSA.

Another area for support identified by participants was a need for more clinical supervision. The benefits of clinical supervision for nurses are well recognized, with evidence to support the effectiveness of both individual and group approaches (McCarthy et al., 2021; Saab et al., 2021). Indeed, staff who work with survivors of CSA have reported that clinical supervision is essential, and that they would struggle with their work without availing of this support (Wheeler & McElvaney, 2018). Despite the benefits afforded to staff, evidence suggests that clinical supervision is not always available, or at the very least, is not consistently available (Capri et al., 2013; Muridzo et al., 2018). Given the importance of supporting survivors of CSA, the negative impact that such engagement can have on psychiatric nurses, and the benefits of clinical supervision, there is a need to embed clinical supervision for staff within the mental health system.

## 6 LIMITATIONS

This study has limitations. A small sample size was utilized, which impacts the generalizability of the study. However, it should be noted that CSA is a challenging topic to talk about, which may have influenced nurses' reluctance to partake in the study. Related to this is another limitation: potential self‐selection bias, meaning those participants who volunteered for the study may have had personal reasons for doing so; those who chose not to volunteer may have had different views on developing therapeutic relationships with survivors of CSA.

## 7 IMPLICATIONS FOR NURSING PRACTICE

Psychiatric nurses have an important role to play in supporting survivors of CSA and developing meaningful therapeutic relationships. Given their facility with interpersonal skills, nurses are ideally positioned to explore trauma and have the potential to positively influence survivors' recovery. However, nurses lack the confidence to employ these skills; consequently, it may not be possible to develop meaningful therapeutic relationships with survivors. It is paramount that interdisciplinary training is provided, with a view towards developing nurses' confidence in exploring trauma and establishing the role of the psychiatric nurse in supporting survivors of CSA. Considering the challenge of discussing traumatic events with survivors and the impact of distressing conversations on the mental health of nurses, health systems need to make more of an effort to provide staff with clinical supervision.

## CONFLICTS OF INTEREST

The authors declare no conflicts of interest.

## Data Availability

data available on request due to privacy/ethical restrictions.
